# (1*R*,4a*S*,10a*R*)-1,4a-Dimethyl-*N*-[(morpholin-4-yl)carbothio­yl]-7-(propan-2-yl)-1,2,3,4,4a,9,10,10a-octa­hydro­phenanthrene-1-carboxamide

**DOI:** 10.1107/S1600536810044569

**Published:** 2010-11-06

**Authors:** Xiao-Ping Rao, Yong Wu, Zhan-Qian Song, Shi-Bin Shang

**Affiliations:** a Institute of Chemical Industry of Forest Products, Chinese Academy of Forestry, Nanjing, 210042, People’s Republic of China; b Zigong Renji Medical Center of Sichuan Province, Zigong, 643000, People’s Republic of China

## Abstract

In the title compound, C_25_H_36_N_2_O_2_S, the cyclo­hexane and morpholine rings adopt chair conformations. The cyclo­hexene and cyclo­hexane rings form a *trans* ring junction with the two methyl groups in axial positions. The N—H and C=O bonds in the urea group are *anti* to each other. The crystal structure is stabilized by inter­molecular N—H⋯O hydrogen bonds.

## Related literature

Dehydro­abietic acid is an abietane diterpenic resin acid which can be easily obtained from Pinus resin or commercial disproportionated rosin, see: Halbrook & Lawrence (1966 ▶). For the biological activity of dehydro­abietic aid derivatives, see: Rao *et al.* (2008 ▶); Sepulveda *et al.* (2005 ▶); Wada *et al.* (1985 ▶); For the crystal structures of dehydro­abietic acid derivatives, see: Rao *et al.* (2006 ▶, 2009 ▶, 2010 ▶).
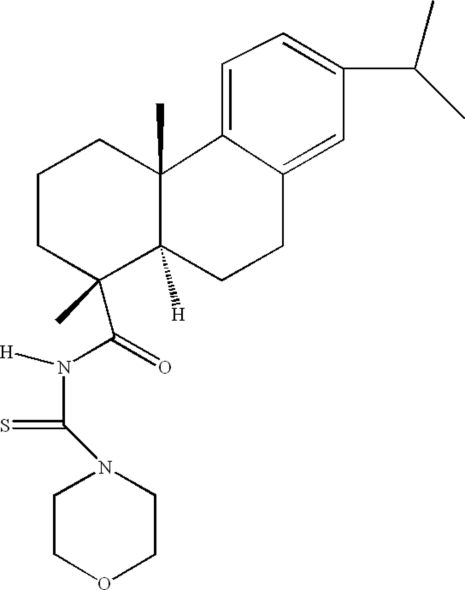


## Experimental

### 

#### Crystal data

C_25_H_36_N_2_O_2_S
*M* *_r_* = 428.62
Orthorhombic, 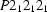

*a* = 9.887 (2) Å
*b* = 15.114 (3) Å
*c* = 16.128 (3) Å
*V* = 2410.0 (8) Å^3^
*Z* = 4
Mo *K*α radiation
μ = 0.16 mm^−1^
*T* = 293 K
0.30 × 0.20 × 0.20 mm

#### Data collection

Entaf–Nonius CAD-4 diffractometer
Absorption correction: ψ scan (North *et al.*, 1968 ▶) *T*_min_ = 0.954, *T*_max_ = 0.969
4802 measured reflections
4370 independent reflections
3137 reflections with *I* > 2σ(*I*)
*R*_int_ = 0.115
3 standard reflections every 200 reflections  intensity decay: 1%

#### Refinement

*R*[*F* ^2^ > 2σ(*F* ^2^)] = 0.069
*wR*(*F* ^2^) = 0.189
*S* = 1.00
4370 reflections
271 parameters
H-atom parameters constrained
Δρ_max_ = 0.37 e Å^−3^
Δρ_min_ = −0.29 e Å^−3^
Absolute structure: Flack (1983 ▶), 1882 Friedel pairs
Flack parameter: −0.08 (16)

### 

Data collection: *CAD-4 Software* (Enraf–Nonius, 1985 ▶); cell refinement: *CAD-4 Software*; data reduction: *XCAD4* (Harms & Wocadlo, 1995 ▶); program(s) used to solve structure: *SHELXS97* (Sheldrick, 2008 ▶); program(s) used to refine structure: *SHELXL97* (Sheldrick, 2008 ▶); molecular graphics: *SHELXTL* (Sheldrick, 2008 ▶); software used to prepare material for publication: *SHELXTL*.

## Supplementary Material

Crystal structure: contains datablocks I, global. DOI: 10.1107/S1600536810044569/bq2238sup1.cif

Structure factors: contains datablocks I. DOI: 10.1107/S1600536810044569/bq2238Isup2.hkl

Additional supplementary materials:  crystallographic information; 3D view; checkCIF report

## Figures and Tables

**Table 1:** Hydrogen-bond geometry (Å, °)

*D*—H⋯*A*	*D*—H	H⋯*A*	*D*⋯*A*	*D*—H⋯*A*
N1—H1*A*⋯O2^i^	0.86	2.45	3.171 (4)	142

Symmetry code: (i) 
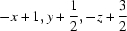
.

**Fig. 1.**
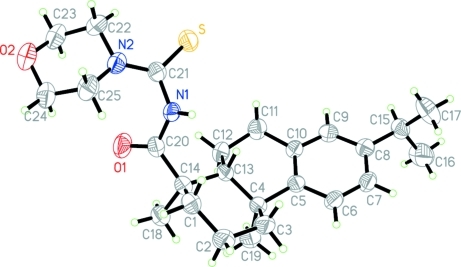
The title compound with displacement ellipsoids at the 30% probability level.

**Fig. 2.**
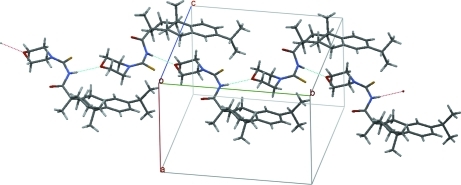
Packing diagrams of title crystal (H atoms omitted for clarity).

## supplementary crystallographic information

### Comment

Dehydroabietic acid is an abietane diterpenic resin acid which can be easily obtained from Pinus resin or commercial disproportionated rosin (Halbrook *et al.*, 1966). Dehydroabietic acid is widely used as starting material for design and synthesis of biological compounds (Sepulveda *et al.*, 2005; Rao *et al.*, 2008; Wada *et al.*, 1985). Crystal structure of dehydroabietic acid derivatives such as acid (Rao *et al.*, 2009), amide (Rao *et al.*, 2006), urea (Rao *et al.*, 2010) were widely investigated. In this work, we describe the crystal structure of the acylthiourea derivative of dehydroabietic acid. Its structure is shown in Figure 1. There are four six-membered rings in the molecule, in which the benzene ring form planar (mean deviation = 0.0055 Å), the cyclohexene ring form half-chair and the cyclohexane and morpholine rings form chair configurations, respectively. The cyclohexene and cyclohexane rings form a *trans* ring junction with two methyl groups in the same side of tricyclo phenanthrene structure. The puckering parameters for the benzene, hexene, hexane and morpholine are [τ = 0.9 °], [(Q) = 0.5384 Å, θ = 48.52 °, φ = 286.4651 °], [(Q) = 0.5530 Å, θ = 176.27 °, φ = 154.5122 °], and [(Q) = 0.5602 Å, θ = 4.25 °, φ = 28.6358 °], respectively. There are three chiral centers in the molecule, they exhibited R–, S– and R– configurations, respectively. The N—H and C=O bonds in the urea group are anti to each other. The crystal structure is stabilized by intermolecular N—H···O hydrogen bonds. The hydrogen bond geometry are listed in Table 1. The packing diagrams of title crystal is shown in Figure 2.

### Experimental

50 mmol dehydroabietyl acylthiourea and 50 mmol morpholine were added to 30 ml dichloromethane, the mixture were refluxed for 6 h, white crystals were obtained after the solvent were distilled off. Single crystals were grown from ethanol.

### Refinement

H atoms were positioned geometrically and refined as riding atoms, with C—H = 0.96Å and *U*_iso_(H) = 1.5*U*_eq_(C) for methyl H atoms, and C—H = 0.97–0.98Å and *U*_iso_(H) = 1.2*U*_eq_(C) for all other H atoms.

### Crystal data

**Table:**

C_25_H_36_N_2_O_2_S	*D*_x_ = 1.181 Mg m^−^^3^
*M**_r_* = 428.62	Melting point: 416 K
Orthorhombic, *P*2_1_2_1_2_1_	Mo *K*α radiation, λ = 0.71073 Å
Hall symbol: P 2ac 2ab	Cell parameters from 25 reflections
*a* = 9.887 (2) Å	θ = 9–12°
*b* = 15.114 (3) Å	µ = 0.16 mm^−^^1^
*c* = 16.128 (3) Å	*T* = 293 K
*V* = 2410.0 (8) Å^3^	Block, white
*Z* = 4	0.30 × 0.20 × 0.20 mm
*F*(000) = 928	

### Data collection

**Table:**

Entaf–Nonius CAD-4 diffractometer	3137 reflections with *I* > 2σ(*I*)
Radiation source: fine-focus sealed tube	*R*_int_ = 0.115
graphite	θ_max_ = 25.3°, θ_min_ = 1.9°
ω/2θ scans	*h* = −11→0
Absorption correction: ψ scan (North *et al.*, 1968)	*k* = −18→0
*T*_min_ = 0.954, *T*_max_ = 0.969	*l* = −19→19
4802 measured reflections	3 standard reflections every 200 reflections
4370 independent reflections	intensity decay: 1%

### Refinement

**Table:**

Refinement on *F*^2^	Secondary atom site location: difference Fourier map
Least-squares matrix: full	Hydrogen site location: inferred from neighbouring sites
*R*[*F*^2^ > 2σ(*F*^2^)] = 0.069	H-atom parameters constrained
*wR*(*F*^2^) = 0.189	*w* = 1/[σ^2^(*F*_o_^2^) + (0.1*P*)^2^ + 1.4*P*] where *P* = (*F*_o_^2^ + 2*F*_c_^2^)/3
*S* = 1.00	(Δ/σ)_max_ < 0.001
4370 reflections	Δρ_max_ = 0.37 e Å^−^^3^
271 parameters	Δρ_min_ = −0.29 e Å^−^^3^
0 restraints	Absolute structure: Flack (1983), 1882 Friedel pairs
Primary atom site location: structure-invariant direct methods	Flack parameter: −0.08 (16)

### Special details

**Table:**

Geometry. All e.s.d.'s (except the e.s.d. in the dihedral angle between two l.s. planes) are estimated using the full covariance matrix. The cell e.s.d.'s are taken into account individually in the estimation of e.s.d.'s in distances, angles and torsion angles; correlations between e.s.d.'s in cell parameters are only used when they are defined by crystal symmetry. An approximate (isotropic) treatment of cell e.s.d.'s is used for estimating e.s.d.'s involving l.s. planes.
Refinement. Refinement of *F*^2^ against ALL reflections. The weighted *R*-factor *wR* and goodness of fit *S* are based on *F*^2^, conventional *R*-factors *R* are based on *F*, with *F* set to zero for negative *F*^2^. The threshold expression of *F*^2^ > σ(*F*^2^) is used only for calculating *R*-factors(gt) *etc*. and is not relevant to the choice of reflections for refinement. *R*-factors based on *F*^2^ are statistically about twice as large as those based on *F*, and *R*- factors based on ALL data will be even larger.

### Fractional atomic coordinates and isotropic or equivalent isotropic displacement parameters (Å^2^)

**Table:**

	*x*	*y*	*z*	*U*_iso_*/*U*_eq_	
S	0.37704 (13)	0.27732 (8)	0.64677 (9)	0.0548 (4)	
N1	0.6164 (4)	0.2465 (2)	0.7135 (2)	0.0375 (8)	
H1A	0.6260	0.2902	0.7471	0.045*	
O1	0.7260 (4)	0.1345 (2)	0.6499 (3)	0.0674 (11)	
C1	0.9067 (5)	0.2895 (3)	0.7578 (3)	0.0512 (12)	
H1B	0.8319	0.3124	0.7902	0.061*	
H1C	0.9378	0.2354	0.7841	0.061*	
O2	0.4399 (4)	−0.0552 (2)	0.7323 (2)	0.0628 (10)	
N2	0.4599 (4)	0.1318 (2)	0.7235 (2)	0.0441 (9)	
C2	1.0214 (5)	0.3570 (3)	0.7579 (3)	0.0544 (13)	
H2A	1.0997	0.3317	0.7305	0.065*	
H2B	1.0465	0.3703	0.8147	0.065*	
C3	0.9808 (5)	0.4423 (3)	0.7140 (3)	0.0483 (12)	
H3A	1.0587	0.4812	0.7120	0.058*	
H3B	0.9115	0.4715	0.7466	0.058*	
C4	0.9273 (4)	0.4294 (3)	0.6252 (3)	0.0385 (10)	
C5	0.8566 (4)	0.5146 (3)	0.5947 (3)	0.0374 (10)	
C6	0.9104 (5)	0.5970 (3)	0.6155 (3)	0.0453 (11)	
H6A	0.9889	0.5994	0.6472	0.054*	
C7	0.8507 (5)	0.6744 (3)	0.5907 (3)	0.0464 (12)	
H7A	0.8884	0.7280	0.6070	0.056*	
C8	0.7352 (4)	0.6744 (3)	0.5417 (3)	0.0403 (10)	
C9	0.6837 (5)	0.5923 (3)	0.5200 (3)	0.0425 (11)	
H9A	0.6071	0.5905	0.4866	0.051*	
C10	0.7401 (4)	0.5129 (3)	0.5454 (3)	0.0374 (10)	
C11	0.6697 (5)	0.4284 (3)	0.5221 (3)	0.0488 (12)	
H11A	0.6509	0.4293	0.4631	0.059*	
H11B	0.5838	0.4256	0.5510	0.059*	
C12	0.7511 (5)	0.3457 (3)	0.5424 (3)	0.0445 (11)	
H12A	0.8203	0.3365	0.5006	0.053*	
H12B	0.6922	0.2944	0.5428	0.053*	
C13	0.8168 (4)	0.3571 (3)	0.6274 (3)	0.0356 (9)	
H13A	0.7453	0.3817	0.6626	0.043*	
C14	0.8569 (4)	0.2682 (3)	0.6697 (3)	0.0435 (11)	
C15	0.6686 (5)	0.7591 (3)	0.5142 (3)	0.0500 (12)	
H15A	0.5932	0.7425	0.4779	0.060*	
C16	0.7624 (6)	0.8168 (4)	0.4634 (5)	0.082 (2)	
H16A	0.7976	0.7833	0.4177	0.123*	
H16B	0.8359	0.8367	0.4976	0.123*	
H16C	0.7134	0.8670	0.4427	0.123*	
C17	0.6084 (7)	0.8099 (4)	0.5869 (4)	0.0810 (19)	
H17A	0.5661	0.8630	0.5670	0.122*	
H17B	0.6789	0.8250	0.6253	0.122*	
H17C	0.5423	0.7737	0.6143	0.122*	
C18	0.9621 (5)	0.2146 (3)	0.6222 (4)	0.0611 (14)	
H18A	0.9813	0.1610	0.6519	0.092*	
H18B	1.0436	0.2487	0.6168	0.092*	
H18C	0.9278	0.2004	0.5682	0.092*	
C19	1.0476 (5)	0.4121 (3)	0.5659 (4)	0.0594 (14)	
H19A	1.0142	0.4034	0.5106	0.089*	
H19B	1.0954	0.3601	0.5835	0.089*	
H19C	1.1077	0.4620	0.5668	0.089*	
C20	0.7293 (5)	0.2095 (3)	0.6764 (3)	0.0438 (11)	
C21	0.4855 (4)	0.2135 (3)	0.6969 (3)	0.0387 (10)	
C22	0.3343 (5)	0.0860 (3)	0.7021 (4)	0.0557 (13)	
H22A	0.2867	0.1187	0.6594	0.067*	
H22B	0.2763	0.0824	0.7505	0.067*	
C23	0.3668 (6)	−0.0061 (3)	0.6713 (3)	0.0589 (14)	
H23A	0.2834	−0.0369	0.6581	0.071*	
H23B	0.4202	−0.0021	0.6210	0.071*	
C24	0.5650 (6)	−0.0118 (3)	0.7501 (4)	0.0636 (15)	
H24A	0.6194	−0.0093	0.7001	0.076*	
H24B	0.6145	−0.0458	0.7911	0.076*	
C25	0.5432 (5)	0.0809 (3)	0.7822 (3)	0.0542 (13)	
H25A	0.4984	0.0785	0.8357	0.065*	
H25B	0.6299	0.1100	0.7896	0.065*	

### Atomic displacement parameters (Å^2^)

**Table:**

	*U*^11^	*U*^22^	*U*^33^	*U*^12^	*U*^13^	*U*^23^
S	0.0510 (7)	0.0422 (6)	0.0712 (9)	0.0040 (6)	−0.0122 (7)	−0.0034 (6)
N1	0.0390 (18)	0.0300 (16)	0.0437 (19)	−0.0022 (15)	0.0017 (17)	−0.0072 (14)
O1	0.069 (2)	0.0348 (17)	0.098 (3)	−0.0044 (17)	0.029 (2)	−0.013 (2)
C1	0.049 (3)	0.042 (2)	0.062 (3)	0.003 (2)	−0.009 (2)	0.013 (2)
O2	0.077 (2)	0.0393 (17)	0.072 (3)	−0.0127 (17)	0.001 (2)	0.0057 (17)
N2	0.048 (2)	0.0376 (19)	0.047 (2)	−0.0062 (17)	0.0031 (19)	−0.0024 (17)
C2	0.046 (3)	0.053 (3)	0.064 (3)	0.000 (2)	−0.014 (3)	0.010 (2)
C3	0.040 (3)	0.046 (3)	0.059 (3)	−0.002 (2)	−0.012 (2)	0.007 (2)
C4	0.033 (2)	0.035 (2)	0.047 (3)	0.0017 (18)	0.0019 (19)	0.0043 (19)
C5	0.035 (2)	0.037 (2)	0.040 (2)	−0.0001 (18)	0.0056 (19)	0.0028 (18)
C6	0.041 (3)	0.043 (2)	0.052 (3)	−0.009 (2)	−0.007 (2)	0.007 (2)
C7	0.049 (3)	0.037 (2)	0.053 (3)	−0.009 (2)	−0.005 (2)	0.003 (2)
C8	0.041 (2)	0.043 (2)	0.037 (3)	−0.002 (2)	0.006 (2)	0.008 (2)
C9	0.042 (2)	0.048 (3)	0.038 (2)	0.002 (2)	−0.005 (2)	0.002 (2)
C10	0.039 (2)	0.036 (2)	0.037 (2)	−0.0033 (19)	−0.001 (2)	0.0014 (18)
C11	0.057 (3)	0.046 (2)	0.044 (3)	−0.003 (2)	−0.010 (2)	−0.004 (2)
C12	0.054 (3)	0.037 (2)	0.042 (3)	−0.006 (2)	0.000 (2)	−0.004 (2)
C13	0.033 (2)	0.032 (2)	0.042 (2)	0.0029 (18)	0.0044 (19)	−0.0002 (18)
C14	0.039 (2)	0.035 (2)	0.056 (3)	0.005 (2)	0.000 (2)	0.003 (2)
C15	0.055 (3)	0.042 (3)	0.053 (3)	0.006 (2)	−0.006 (2)	0.008 (2)
C16	0.074 (4)	0.071 (4)	0.102 (5)	0.008 (3)	0.004 (4)	0.041 (4)
C17	0.092 (4)	0.076 (4)	0.075 (4)	0.038 (4)	0.004 (4)	−0.013 (3)
C18	0.048 (3)	0.043 (3)	0.093 (4)	0.006 (2)	0.014 (3)	−0.001 (3)
C19	0.042 (3)	0.056 (3)	0.081 (4)	0.000 (2)	0.018 (3)	0.008 (3)
C20	0.047 (3)	0.033 (2)	0.052 (3)	0.004 (2)	0.002 (2)	0.004 (2)
C21	0.043 (2)	0.035 (2)	0.038 (2)	0.002 (2)	0.004 (2)	−0.0102 (19)
C22	0.046 (3)	0.049 (3)	0.072 (4)	−0.012 (2)	0.007 (3)	0.001 (3)
C23	0.072 (3)	0.045 (3)	0.060 (3)	−0.017 (3)	0.007 (3)	−0.002 (2)
C24	0.070 (3)	0.040 (3)	0.080 (4)	−0.004 (2)	−0.004 (3)	0.015 (3)
C25	0.068 (3)	0.051 (3)	0.043 (3)	−0.009 (3)	0.000 (3)	0.013 (2)

### Geometric parameters (Å, °)

**Table:**

S—C21	1.654 (5)	C11—H11A	0.9700
N1—C20	1.384 (6)	C11—H11B	0.9700
N1—C21	1.412 (6)	C12—C13	1.526 (6)
N1—H1A	0.8600	C12—H12A	0.9700
O1—C20	1.213 (5)	C12—H12B	0.9700
C1—C2	1.524 (6)	C13—C14	1.559 (6)
C1—C14	1.539 (7)	C13—H13A	0.9800
C1—H1B	0.9700	C14—C18	1.524 (6)
C1—H1C	0.9700	C14—C20	1.546 (6)
O2—C24	1.429 (6)	C15—C16	1.514 (8)
O2—C23	1.429 (6)	C15—C17	1.523 (8)
N2—C21	1.330 (5)	C15—H15A	0.9800
N2—C22	1.464 (6)	C16—H16A	0.9600
N2—C25	1.472 (6)	C16—H16B	0.9600
C2—C3	1.525 (6)	C16—H16C	0.9600
C2—H2A	0.9700	C17—H17A	0.9600
C2—H2B	0.9700	C17—H17B	0.9600
C3—C4	1.538 (7)	C17—H17C	0.9600
C3—H3A	0.9700	C18—H18A	0.9600
C3—H3B	0.9700	C18—H18B	0.9600
C4—C13	1.545 (6)	C18—H18C	0.9600
C4—C5	1.546 (6)	C19—H19A	0.9600
C4—C19	1.549 (6)	C19—H19B	0.9600
C5—C6	1.395 (6)	C19—H19C	0.9600
C5—C10	1.399 (6)	C22—C23	1.513 (7)
C6—C7	1.370 (6)	C22—H22A	0.9700
C6—H6A	0.9300	C22—H22B	0.9700
C7—C8	1.389 (6)	C23—H23A	0.9700
C7—H7A	0.9300	C23—H23B	0.9700
C8—C9	1.386 (6)	C24—C25	1.509 (7)
C8—C15	1.506 (6)	C24—H24A	0.9700
C9—C10	1.386 (6)	C24—H24B	0.9700
C9—H9A	0.9300	C25—H25A	0.9700
C10—C11	1.502 (6)	C25—H25B	0.9700
C11—C12	1.523 (6)		
			
C20—N1—C21	121.0 (3)	C18—C14—C1	110.9 (4)
C20—N1—H1A	119.5	C18—C14—C20	106.7 (4)
C21—N1—H1A	119.5	C1—C14—C20	108.4 (4)
C2—C1—C14	112.2 (4)	C18—C14—C13	114.3 (4)
C2—C1—H1B	109.2	C1—C14—C13	107.8 (3)
C14—C1—H1B	109.2	C20—C14—C13	108.5 (3)
C2—C1—H1C	109.2	C8—C15—C16	112.4 (4)
C14—C1—H1C	109.2	C8—C15—C17	111.8 (4)
H1B—C1—H1C	107.9	C16—C15—C17	111.5 (5)
C24—O2—C23	109.7 (4)	C8—C15—H15A	106.9
C21—N2—C22	121.6 (4)	C16—C15—H15A	106.9
C21—N2—C25	125.9 (4)	C17—C15—H15A	106.9
C22—N2—C25	112.3 (4)	C15—C16—H16A	109.5
C1—C2—C3	111.7 (4)	C15—C16—H16B	109.5
C1—C2—H2A	109.3	H16A—C16—H16B	109.5
C3—C2—H2A	109.3	C15—C16—H16C	109.5
C1—C2—H2B	109.3	H16A—C16—H16C	109.5
C3—C2—H2B	109.3	H16B—C16—H16C	109.5
H2A—C2—H2B	107.9	C15—C17—H17A	109.5
C2—C3—C4	114.6 (4)	C15—C17—H17B	109.5
C2—C3—H3A	108.6	H17A—C17—H17B	109.5
C4—C3—H3A	108.6	C15—C17—H17C	109.5
C2—C3—H3B	108.6	H17A—C17—H17C	109.5
C4—C3—H3B	108.6	H17B—C17—H17C	109.5
H3A—C3—H3B	107.6	C14—C18—H18A	109.5
C3—C4—C13	108.2 (4)	C14—C18—H18B	109.5
C3—C4—C5	110.2 (3)	H18A—C18—H18B	109.5
C13—C4—C5	106.0 (3)	C14—C18—H18C	109.5
C3—C4—C19	109.4 (4)	H18A—C18—H18C	109.5
C13—C4—C19	115.9 (4)	H18B—C18—H18C	109.5
C5—C4—C19	106.9 (4)	C4—C19—H19A	109.5
C6—C5—C10	117.8 (4)	C4—C19—H19B	109.5
C6—C5—C4	119.6 (4)	H19A—C19—H19B	109.5
C10—C5—C4	122.5 (4)	C4—C19—H19C	109.5
C7—C6—C5	121.9 (4)	H19A—C19—H19C	109.5
C7—C6—H6A	119.1	H19B—C19—H19C	109.5
C5—C6—H6A	119.1	O1—C20—N1	120.6 (4)
C6—C7—C8	121.4 (4)	O1—C20—C14	122.2 (4)
C6—C7—H7A	119.3	N1—C20—C14	117.2 (4)
C8—C7—H7A	119.3	N2—C21—N1	116.1 (4)
C9—C8—C7	116.4 (4)	N2—C21—S	125.2 (3)
C9—C8—C15	121.7 (4)	N1—C21—S	118.7 (3)
C7—C8—C15	121.9 (4)	N2—C22—C23	109.4 (4)
C10—C9—C8	123.5 (4)	N2—C22—H22A	109.8
C10—C9—H9A	118.2	C23—C22—H22A	109.8
C8—C9—H9A	118.2	N2—C22—H22B	109.8
C9—C10—C5	118.9 (4)	C23—C22—H22B	109.8
C9—C10—C11	118.4 (4)	H22A—C22—H22B	108.2
C5—C10—C11	122.6 (4)	O2—C23—C22	111.1 (4)
C10—C11—C12	113.5 (4)	O2—C23—H23A	109.4
C10—C11—H11A	108.9	C22—C23—H23A	109.4
C12—C11—H11A	108.9	O2—C23—H23B	109.4
C10—C11—H11B	108.9	C22—C23—H23B	109.4
C12—C11—H11B	108.9	H23A—C23—H23B	108.0
H11A—C11—H11B	107.7	O2—C24—C25	111.8 (4)
C11—C12—C13	109.0 (3)	O2—C24—H24A	109.2
C11—C12—H12A	109.9	C25—C24—H24A	109.2
C13—C12—H12A	109.9	O2—C24—H24B	109.2
C11—C12—H12B	109.9	C25—C24—H24B	109.2
C13—C12—H12B	109.9	H24A—C24—H24B	107.9
H12A—C12—H12B	108.3	N2—C25—C24	110.2 (4)
C12—C13—C4	111.2 (4)	N2—C25—H25A	109.6
C12—C13—C14	113.8 (3)	C24—C25—H25A	109.6
C4—C13—C14	116.1 (3)	N2—C25—H25B	109.6
C12—C13—H13A	104.8	C24—C25—H25B	109.6
C4—C13—H13A	104.8	H25A—C25—H25B	108.1
C14—C13—H13A	104.8		
			
C14—C1—C2—C3	56.3 (6)	C2—C1—C14—C18	70.6 (5)
C1—C2—C3—C4	−54.2 (6)	C2—C1—C14—C20	−172.5 (4)
C2—C3—C4—C13	50.3 (5)	C2—C1—C14—C13	−55.2 (5)
C2—C3—C4—C5	165.9 (4)	C12—C13—C14—C18	62.4 (5)
C2—C3—C4—C19	−76.8 (5)	C4—C13—C14—C18	−68.6 (5)
C3—C4—C5—C6	38.4 (5)	C12—C13—C14—C1	−173.9 (4)
C13—C4—C5—C6	155.3 (4)	C4—C13—C14—C1	55.2 (5)
C19—C4—C5—C6	−80.5 (5)	C12—C13—C14—C20	−56.6 (5)
C3—C4—C5—C10	−142.6 (4)	C4—C13—C14—C20	172.5 (4)
C13—C4—C5—C10	−25.7 (5)	C9—C8—C15—C16	−120.9 (5)
C19—C4—C5—C10	98.6 (5)	C7—C8—C15—C16	59.7 (7)
C10—C5—C6—C7	1.5 (7)	C9—C8—C15—C17	112.8 (6)
C4—C5—C6—C7	−179.4 (4)	C7—C8—C15—C17	−66.6 (6)
C5—C6—C7—C8	−1.6 (7)	C21—N1—C20—O1	−22.3 (7)
C6—C7—C8—C9	0.4 (7)	C21—N1—C20—C14	157.0 (4)
C6—C7—C8—C15	179.8 (5)	C18—C14—C20—O1	2.7 (7)
C7—C8—C9—C10	0.9 (7)	C1—C14—C20—O1	−116.8 (5)
C15—C8—C9—C10	−178.6 (4)	C13—C14—C20—O1	126.4 (5)
C8—C9—C10—C5	−0.9 (7)	C18—C14—C20—N1	−176.5 (4)
C8—C9—C10—C11	176.2 (4)	C1—C14—C20—N1	64.0 (5)
C6—C5—C10—C9	−0.3 (6)	C13—C14—C20—N1	−52.8 (5)
C4—C5—C10—C9	−179.4 (4)	C22—N2—C21—N1	−172.8 (4)
C6—C5—C10—C11	−177.3 (4)	C25—N2—C21—N1	13.3 (6)
C4—C5—C10—C11	3.6 (6)	C22—N2—C21—S	7.1 (6)
C9—C10—C11—C12	171.6 (4)	C25—N2—C21—S	−166.8 (4)
C5—C10—C11—C12	−11.3 (6)	C20—N1—C21—N2	66.4 (5)
C10—C11—C12—C13	41.6 (5)	C20—N1—C21—S	−113.5 (4)
C11—C12—C13—C4	−68.1 (5)	C21—N2—C22—C23	131.5 (4)
C11—C12—C13—C14	158.6 (4)	C25—N2—C22—C23	−53.8 (5)
C3—C4—C13—C12	175.6 (4)	C24—O2—C23—C22	−61.0 (5)
C5—C4—C13—C12	57.4 (4)	N2—C22—C23—O2	58.0 (6)
C19—C4—C13—C12	−61.1 (5)	C23—O2—C24—C25	59.5 (6)
C3—C4—C13—C14	−52.2 (5)	C21—N2—C25—C24	−133.2 (5)
C5—C4—C13—C14	−170.5 (4)	C22—N2—C25—C24	52.4 (5)
C19—C4—C13—C14	71.1 (5)	O2—C24—C25—N2	−54.9 (6)

### Hydrogen-bond geometry (Å, °)

**Table:**

*D*—H···*A*	*D*—H	H···*A*	*D*···*A*	*D*—H···*A*
N1—H1A···O2^i^	0.86	2.45	3.171 (4)	142

Symmetry codes: (i) −*x*+1, *y*+1/2, −*z*+3/2.
